# Impact of Situation Awareness Variations on Multimodal Physiological Responses in High-Speed Train Driving

**DOI:** 10.3390/brainsci14111156

**Published:** 2024-11-20

**Authors:** Wenli Dong, Weining Fang, Hanzhao Qiu, Haifeng Bao

**Affiliations:** 1 State Key Laboratory of Advanced Rail Autonomous Operation, Beijing Jiaotong University, Beijing 100044, China; wldong@bjtu.edu.cn (W.D.); hfbao@bjtu.edu.cn (H.B.); 2 School of Automation and Intelligence, Beijing Jiaotong University, Beijing 100044, China; 3 School of Mechanical, Electronic and Control Engineering, Beijing Jiaotong University, Beijing 100044, China; hzhqiu@bjtu.edu.cn

**Keywords:** situation awareness (SA), multimodal physiological signals, EEG, high-speed train driving, SA labeling methods, SA measurement paradigms

## Abstract

Background: In safety-critical environments, human error is a leading cause of accidents, with the loss of situation awareness (SA) being a key contributing factor. Accurate SA assessment is essential for minimizing such risks and ensuring operational safety. Traditional SA measurement methods have limitations in dynamic real-world settings, while physiological signals, particularly EEG, offer a non-invasive, real-time alternative for continuous SA monitoring. However, the reliability of SA measurement based on physiological signals depends on the accuracy of SA labeling. Objective: This study aims to design an effective SA measurement paradigm specific to high-speed train driving, investigate more accurate physiological signal-based SA labeling methods, and explore the relationships between SA levels and key physiological metrics based on the developed framework. Methods: This study recruited 19 male high-speed train driver trainees and developed an SA measurement paradigm specific to high-speed train driving. A method combining subjective SA ratings and task performance was introduced to generate accurate SA labels. Results: The results of statistical analysis confirmed the effectiveness of this paradigm in inducing SA level changes, revealing significant relationships between SA levels and key physiological metrics, including eye movement patterns, ECG features (e.g., heart rate variability), and EEG power spectral density across theta, alpha, and beta bands. Conclusions: This study supports the use of multimodal physiological signals for SA assessment and provides a theoretical foundation for future applications of SA monitoring in railway operations, contributing to enhanced operational safety.

## 1. Introduction

Human error has been widely recognized as a major contributor to accidents in safety-critical domains [1], such as aviation, nuclear power, and rail transportation. It is estimated that 60% to 80% of aviation accidents can be attributed to human error [2,3,4]. Notable incidents like the Three Mile Island and Chernobyl nuclear disasters were also closely tied to human error [5,6]. In rail transportation, safety reports from the International Union of Railways (UIC) between 2007 and 2020 consistently identified human error as a leading cause of railway accidents [7].

One of the key factors contributing to human error is poor situation awareness (SA) [8], which encompasses the perception, understanding, and projection of events in a given environment [9]. In fact, SA-related issues have been identified as contributing factors in 88% of airline incidents involving human error [10]. In the context of high-speed rail, SA is essential for train drivers to effectively monitor and respond to a dynamic operating environment. Train drivers are required to maintain awareness of both the internal state of the cabin and external conditions, which include monitoring speed, interpreting signals, assessing weather conditions, and identifying potential obstacles on the track [11,12,13,14]. Good SA enables drivers to detect and assess potential hazards quickly, make informed decisions, plan appropriate responses, and execute actions effectively, thus minimizing the likelihood of accidents [13,15,16]. Conversely, poor SA is closely associated with human errors in train driving, such as failing to notice critical signals, misinterpreting speed limits, or overlooking cabin alarms, which can lead to overspeeding, delayed braking, and other operational risks [17]. Given the critical role that SA plays in preventing accidents in high-speed rail, it is essential to detect SA loss and implement timely interventions to enhance operational safety.

SA measurement methods are typically divided into direct and indirect approaches. Direct methods include techniques such as the Situation Awareness Global Assessment Technique (SAGAT), real-time probes, post-task self-ratings, and observer ratings [18]. While these methods provide valuable insights into SA, their application in dynamic operational settings is limited, particularly due to the disruptive nature of task freezes required by SAGAT [19]. In contrast, indirect methods infer SA through performance data or process indicators. These approaches can monitor SA in real time without interrupting the task [20]. However, inferring SA based solely on performance assumes that good task performance equates to high SA, which is not always accurate [18]. Physiological signals, as the main part of the process indicators, offer the potential for continuous, real-time, and non-invasive SA measurement and demonstrate considerable promise for long-term SA monitoring in real-world environments.

Current research has identified various physiological signals related to SA, including eye-tracking (ET), electrocardiography (ECG), and electroencephalography (EEG) [18]. However, there is no consensus on the optimal physiological signal for assessing SA. Each signal provides valuable but limited insights into different aspects of SA. Many studies have utilized eye-tracking data to evaluate SA, focusing primarily on fixation-related metrics such as fixation rate, fixation count, dwell time, and dwell count. These metrics have been found to positively correlate with SA levels [21,22,23,24], as they serve as indicators of visual attention, which is closely associated with Level 1 SA (perception). However, ET is prone to limitations like the “look-but-don’t-see” phenomenon [20], where drivers may visually attend to an area without fully processing the information, leading to potential gaps in SA. ECG, which reflects autonomic nervous system activity, has shown correlations between SA and certain metrics, such as heart rate (HR) [25,26] and heart rate variability (HRV) features [27,28,29]. Specifically, time-domain HRV features like the root mean square of successive differences (RMSSD) have been found to positively correlate with SA [28], providing an additional measure of SA state changes. However, ECG signals can be highly sensitive to external conditions and task demands, which may introduce variability in operational environments [18]. EEG, capable of capturing all three levels of SA (perception, comprehension, and projection), provides direct measures of brain activity. Research has demonstrated that EEG features are associated with different SA states, with specific frequency bands reflecting neural activity linked to SA [30,31,32]. While EEG offers valuable insights, it also faces challenges such as complexity, noise, and non-stationarity [18], which can complicate its application. Given the limitations of individual signals, combining multiple physiological signals offers a more comprehensive understanding of SA dynamics, thereby improving the accuracy of SA inference.

To ensure accurate SA evaluation using physiological signals, researchers typically rely on direct SA measurements and performance-based SA assessments to establish the “ground truth” for labeling physiological data. The three primary methods currently used for SA labeling are SAGAT [30,33,34], the Situation Awareness Rating Technique (SART, a subjective self-report) [31], and performance-based metrics [35,36,37]. Although SAGAT provides precise and direct SA assessments, its task-freezing requirement limits its applicability in real-world environments. Subjective ratings, while easy to implement, often reflect operators’ confidence rather than their actual SA [38]. Performance-based metrics assume that high task performance corresponds to high SA, which may not always hold true [18]. Therefore, developing more accurate SA labeling methods for physiological signals is crucial for improving the reliability of SA evaluation based on physiological data [39].

In prior SA studies, two main paradigms for measuring SA have emerged [18]. The first uses SA as a metric to evaluate system design [40], where system factors such as interface layout or visual indicators affect operators’ ability to perceive and understand the environment, ultimately influencing SA level. In this paradigm, the relationship between direct SA measurements and physiological signals is often indirectly constructed through third-party factors such as task performance. The second paradigm directly examines the relationship between SA indicators and physiological signals [37], typically using a single task as a representative research subject. In this approach, SA differences are often attributed to variations in context or individual differences. Given that the factors influencing SA variations differ across applications, it is necessary to design effective SA measurement paradigms specific to the application background. This involves selecting appropriate tasks to represent the research subject and exploring methods to induce SA changes. Doing so can provide a reliable data foundation for studying the relationship between SA and multimodal physiological signals, ultimately enhancing the reliability and applicability of SA-related physiological features.

In summary, this study aims to develop an SA measurement paradigm tailored to the high-speed train driving context and, by combining subjective SA ratings and task performance metrics, to develop more accurate SA labeling methods. Based on these, we will explore the relationship between SA changes and multimodal physiological signal features. We hypothesize that the SA measurement paradigm developed for the high-speed train driving context will effectively induce variations in SA states, and these SA changes will be reflected in distinct physiological patterns, as evidenced by significant differences in typical physiological signal features across different SA levels. The results will provide theoretical support and an experimental foundation for developing physiological signal-based SA measurement methods for high-speed train drivers and lay the groundwork for future applications of multimodal SA monitoring in this field.

## 2. Materials and Methods

### 2.1. Subjects

We recruited 19 male train driver trainees aged 19 to 22 years (mean age = 20.5 years, standard deviation = 0.8 years) from Zhengzhou Railway Vocational and Technical College. Thus, they were a homogenous group in terms of sex and age in order to minimize potential negative impact of these variables on SA estimation accuracy. These subjects had normal or corrected-to-normal vision and no color deficiency, color blindness, hearing impairments, physical limitations, or other relevant impairments. The study protocol obtained approval from the Ethical Committee of Beijing Jiaotong University, and all subjects provided informed consent prior to their participation.

### 2.2. Apparatus

**Figure 1:**
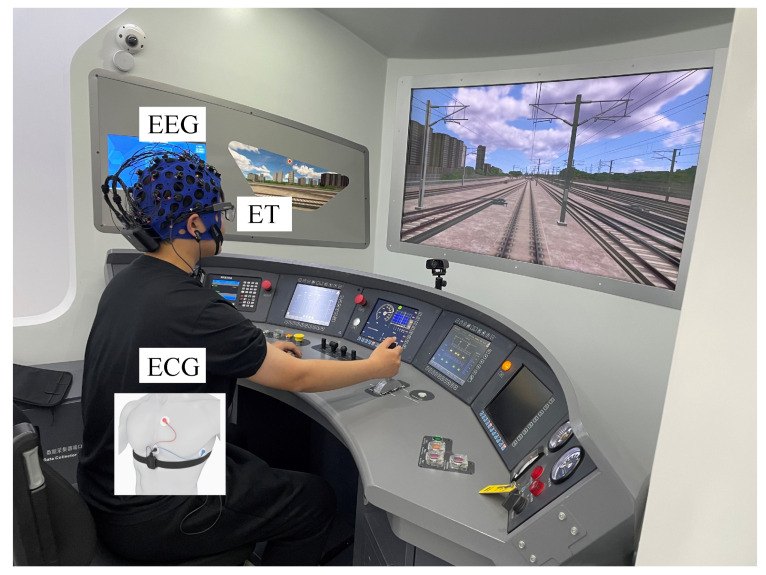
Experimental environment for signals acquisition under a simulated driving environment.

The study was conducted using a driving simulator that simulates the CR400BF Chinese standard EMU (Electric Multiple Unit). The simulator was manufactured by Think Freely Hi-Tech Co., Ltd. (Zhengzhou, China). ET data were captured using the Tobii Pro Glasses 3, with a sampling rate of 15 Hz. A 32-channel BitBrain semi-dry EEG system was employed, operating at a sampling rate of 256 Hz. ECG signals were recorded using a wearable physiological signal recording system with a sampling rate of 512 Hz. The simulated driving environment and data acquisition setup are illustrated in Figure 1.

**Figure 2:**
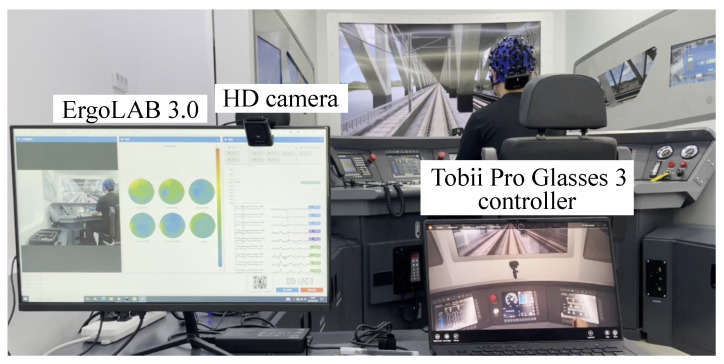
Multimodal data acquisition and synchronization.

Real-time synchronization of EEG and ECG data was achieved using ErgoLAB (v3.0) software. After the experiment, eye-tracking data were imported into ErgoLAB, where temporal synchronization with the other three sensor modalities was performed by comparing the recorded high-definition camera footage with the ET video. The multimodal data acquisition and synchronization setup is shown in Figure 2.

### 2.3. Experimental Tasks

The experiment involved four primary train driving tasks: environmental monitoring, speed adjustment, fault detection, and fault diagnosis. All participants operated the train on the same route, maintaining vigilance on the environment, tracking the target speed, and ensuring it did not exceed the permitted limits. Randomly occurring fault events or abnormal driving events were introduced during the simulation. Participants were required to respond promptly to these events.

The experiment included four fault events and four abnormal driving events. The fault events consisted of a vacuum circuit breaker that cannot be closed, traction loss—type 1, traction loss—type 2, and auxiliary converter not working. The abnormal driving events involved driving in rainy weather (onset and conclusion) and driving in foggy weather (onset and conclusion). A wireless communication interface was developed to synchronize the simulator with the ErgoLAB (v3.0) software, ensuring accurate recording of the event markers ‘event occurrence’ and ‘subject response’.

### 2.4. Experimental Design

The experiment utilized a within-subject design, where each participant completed tasks under four experimental conditions in a randomized order. The four experimental conditions were structured as follows:

(1) Condition 1: Neither fatigue nor stress was induced.
(2) Condition 2: Stress was induced without inducing fatigue.
(3) Condition 3: Fatigue was induced without inducing stress.
(4) Condition 4: Both fatigue and stress were induced.

Fatigue was induced by having participants perform a 90-min AX-CPT (AX version of Continuous Performance Task) paradigm, which has been shown to be effective in inducing fatigue [41,42]. Stress was induced by imposing a speed requirement during a simulated driving task, where participants were instructed to keep the difference between their current speed and the target speed within 10 km/h while ensuring they did not exceed the speed limit.

To assess the subjects’ mental states, three subjective scales were employed: the Karolinska Sleepiness Scale (KSS), a widely used 9-point scale for measuring fatigue level [43], ranging from “extremely alert” to “extremely sleepy”; the State Anxiety Inventory-6 (SATI-6) [44], a brief 6-item questionnaire designed to evaluate the intensity of stress; and an adapted version of the Mission Awareness Rating Scale (MARS) [45,46], originally developed for pilots, which we modified to suit the context of train driving tasks (see Appendix A for details).

### 2.5. Procedure

Before initiating the experiment, subjects were briefed on the study’s objectives and provided informed consent. Practice sessions were then conducted to familiarize subjects with the response procedures for fault and abnormal driving events. Following the practice sessions, subjects prepared for the simulated driving task, including equipment calibration and testing.

**Figure 3:**
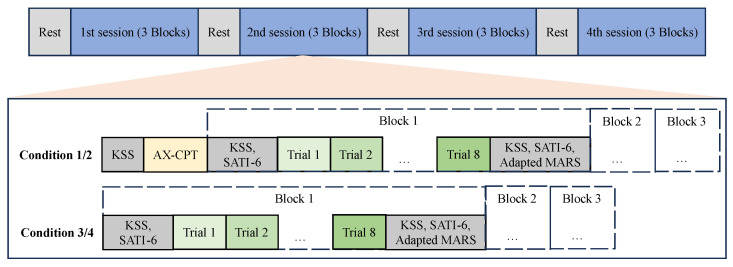
Experimental procedure.

During the formal experiment, each condition (session) was divided into 3 blocks, with each block containing 8 trials, corresponding to 8 events, as shown in Figure 3. The sequence of events within each block was randomized using a Latin Square design to minimize order effects. Additionally, the order of the blocks was counterbalanced across subjects to ensure balanced exposure and control for potential learning effects.

## 3. Methodology

### 3.1. Determination of Mental State Labels

The determination of fatigue, stress, and SA labels was structured as follows:

(1) Determination of Fatigue Label: KSS was used to determine fatigue levels. Scores ranging from 1 to 5 were classified as low fatigue, while scores from 6 to 9 were classified as high fatigue.
(2) Determination of Stress Label: Stress levels were determined using the State Anxiety Inventory-6 (SATI-6). Scores were dichotomized into two categories: high stress (scores≥15) and low stress (scores<15).
(3) Determination of SA Label: To generate more accurate SA labels, both subjective SA scores and response time (RT) were utilized. A Gaussian Mixture Model (GMM) was employed for clustering RT, leveraging its flexibility and probabilistic framework, which makes it a powerful tool for clustering tasks with known class numbers. RT was clustered into two components, with the intersection of the probability density functions of these components serving as the threshold to distinguish between high and low SA. For subjective SA scores, the median was used as the threshold to categorize SA levels. To avoid interference with the driving task, subjective SA ratings were collected at the end of each block, which consisted of eight SA events. Given the potential for RT to more objectively reflect SA, RT was prioritized in determining the final SA label. The labeling rules were as follows: if the RT exceeded the threshold, the data point was labeled as low SA; if the RT did not exceed the threshold but the subjective SA score exceeded the threshold, the data point was also labeled as low SA; if neither condition was met, the data point was labeled as high SA.

### 3.2. Validation of Fatigue and Stress Induction

The effectiveness of the fatigue and stress induction methods was validated through subjective measurements collected at key points during the experiment, as depicted in Figure 3. Linear mixed models were applied to analyze changes in subjective fatigue and stress scores over time, providing statistical validation of the induction methods. To validate the effectiveness of the SA induction methods, a Chi-square test was conducted to assess the distribution of high and low SA samples across different experimental conditions.

### 3.3. Signal Preprocessing

First, data segments were extracted from the period between the “event occurrence” and the “subject response”, with variable lengths depending on individual reaction times. A total of 1824 data segments were collected (19 subjects × 4 conditions × 3 blocks × 8 trials). After removing invalid segments, 1062 valid samples were retained for further analysis.

Next, the multimodal physiological signals (ET, ECG, EEG) were preprocessed to improve signal quality and remove artifacts:

(1) ET: For ET data, missing data segments up to 75 ms were linearly interpolated to improve data continuity. Noise reduction was achieved using a sliding median filter with a window size of 3 samples. Eye movements were classified into saccades and fixations using the Velocity-Threshold Identification algorithm, with an angular velocity threshold set to 30°/s. Samples with velocities exceeding this threshold were classified as saccades, while samples below the threshold were classified as fixations.
(2) ECG: ECG signals were preprocessed by applying a band-pass filter (0.05–100 Hz) to remove baseline drift and high-frequency noise. Further denoising was performed using wavelet decomposition, specifically designed to retain essential ECG features while reducing transient noise. The R-peaks were identified using a peak detection algorithm optimized for the sampling rate of 512 Hz. We ensured the accuracy of R-peak detection by visually inspecting a subset of data and manually correcting any misdetections.
(3) EEG: For EEG data, initial preprocessing involved identifying and interpolating bad channels based on criteria such as high variance or flat-line signals, using spherical spline interpolation. EEG signals were then re-referenced to the common average reference (CAR) and band-pass filtered between 1 and 30 Hz to retain relevant frequency bands (theta, alpha, and beta) while reducing low-frequency drift and high-frequency noise. Independent Component Analysis (ICA) was performed to separate and remove artifacts associated with eye blinks, muscle movements, and cardiac signals. Typically, 1–3 components were removed per subject based on visual inspection of the component topographies and time courses to ensure the retention of neural signals while removing non-neural artifacts.

### 3.4. Feature Extraction

In this study, typical features were extracted from two physiological signals: ECG and EEG. Eye-tracking data were analyzed through heatmaps rather than specific feature extraction to understand attention distribution and its relation to SA.

(1) ECG Features

A total of 9 features were extracted from the ECG signals, focusing on heart rate variability (HRV) metrics across time-domain and frequency-domain measures. Time-domain features included Mean HR (bpm), SDNN (ms), RMSSD (ms), SDSD (ms), and pNN50 (%). Frequency-domain features included LF Power (ms^2^), HF Power (ms^2^), Total Power (ms^2^), and the LF/HF ratio. These features were chosen to provide insights into autonomic nervous system activity and its relationship with SA.

Given that the length of each data segment varies between 30 s and 1 min, we applied a 30-s window with 50% overlap to each segment for frequency-domain HRV analysis. This approach has been shown to be effective for ultra-short-term HRV analysis, where shorter windows may not capture stable frequency-domain features [47,48]. Specifically, the 30-s window length is commonly recommended as the minimum for capturing reliable LF and HF power components, especially in short-duration recordings [49]. The 50% overlap helps to increase the effective data length, thereby enhancing the stability of frequency-domain calculations in short segments [47]. This setup ensures sufficient data length within each window to capture reliable frequency components, addressing the variability in segment length while maintaining the stability of the LF and HF power calculations.

(2) EEG Features

EEG power spectral density (PSD) features were extracted across three frequency bands: theta (4–8 Hz), alpha (8–13 Hz), and beta (13–30 Hz). PSD is utilized as the frequency-domain feature because it is the most commonly used physiological indicator of SA in EEG studies [20,50]. PSD provides a measure of the power distribution of the EEG signal across different frequency bands, which is critical for understanding the underlying neural oscillations associated with various cognitive states. By analyzing the PSD, we can gain insights into the strength of oscillations at specific frequencies, which are linked to different brain functions and psychological states.

These specific frequency bands were selected due to their well-documented associations with cognitive processing. Theta band activity, particularly in the frontal cortex, is known to relate to working memory and attentional control, which are essential for SA in dynamic tasks [51,52]. Alpha oscillations in the parietal and occipital regions are linked to attentional modulation and cortical inhibition, with decreases in alpha power reflecting increased cognitive effort and heightened attention to task-relevant stimuli [53,54]. Beta band activity, especially in the parietal and frontal regions, is associated with cognitive engagement, attentional control, and task-focused processing, which are essential for maintaining stable cognitive processes in demanding environments [55,56].

Given the variable length of each data segment (from “event occurrence” to “subject response”), we further divided each segment into non-overlapping 1-s epochs to ensure consistency in spectral analysis. For each segment, we computed the PSD for each 1-s epoch. The final PSD value for each segment was obtained by averaging the PSD values of all 1-s epochs within that segment. Using this method allowed us to account for the variability in segment length while maintaining a consistent epoch size for PSD calculation, which is critical for reliable frequency-domain analysis. The EEG data was recorded from 32 electrodes, with PSD features computed for each electrode across the three frequency bands, resulting in a total of 96 EEG features (1 PSD feature × 32 electrodes × 3 frequency bands).

## 4. Results

### 4.1. SA Labeling Using Response Time and Subjective Ratings

**Figure 4:**
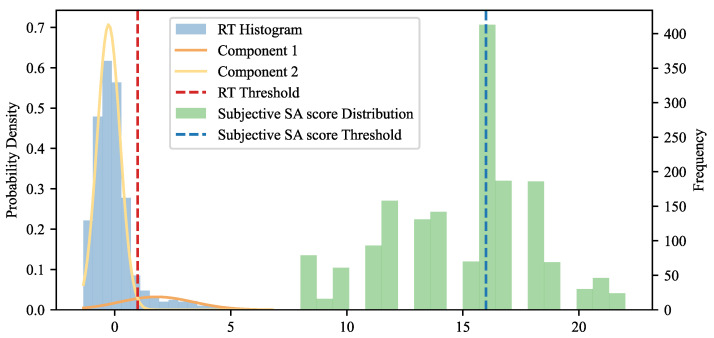
Two-component GMM is fitted on RT and median of SA subjective score is used to identify the threshold of high and low SA.

The classification of SA was based on both response time (RT) and subjective SA ratings. RT data were standardized, and the threshold for distinguishing high and low SA was determined by the intersection of the two components’ probability density functions from GMM. As shown in Figure 4, this intersection, at a normalized value of 0.99, corresponds to an actual RT of 6.55 s. For subjective SA scores, the median value of 16 was used as the classification threshold.

To minimize interference with the driving task, subjective SA ratings were collected at the end of each block, which consisted of eight SA events. Given the real-time nature of RT, it was prioritized in SA classification. The data labeling process followed these rules: If RT exceeded the threshold, regardless of the subjective SA score, the data point was labeled as low SA. If RT was below the threshold but the subjective SA score exceeded 16, the data point was also labeled as low SA. Otherwise, it was labeled as high SA. In total, 1062 samples were classified as high SA, and 643 were classified as low SA.

### 4.2. Validation of Fatigue and Stress Induction

**Figure 5:**
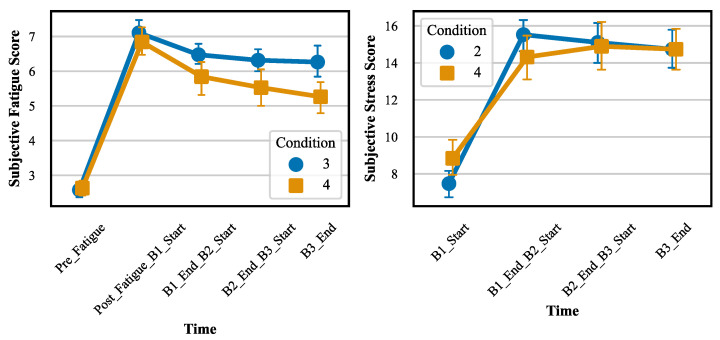
Trends in subjective fatigue and stress scores over time for different experimental conditions.

The effectiveness of the fatigue and stress induction methods was confirmed through both visual analysis of subjective fatigue and stress scores over time, as well as statistical testing. Figure 5 presents the combined trends for subjective fatigue and stress scores across time.

In terms of fatigue induction, the subjective fatigue scores showed a significant increase after the AX-CPT task, followed by a gradual decrease across the experimental blocks. The linear mixed model analysis revealed a significant main effect of time on subjective fatigue scores (F(5,90)=10.21,p<0.001). Post hoc comparisons using the Bonferroni correction confirmed that subjective fatigue scores were significantly higher at Post_Fatigue_B1_Start compared to baseline (p<0.001). Although fatigue levels decreased throughout the experiment, they remained significantly elevated compared to baseline at all time points (p<0.05).

Regarding stress induction, subjective stress scores significantly increased after the stress-inducing task and remained relatively stable across the experimental blocks. The linear mixed model analysis indicated a significant main effect of time on subjective stress scores (F(4,72)=13.55,p<0.001). Post hoc comparisons revealed a significant increase in stress scores from B1_Start to B1_End_B2_Start (p<0.001), with stress levels remaining elevated for the remainder of the experiment.

### 4.3. Validation of SA Induction

The distribution of high and low SA samples under different experimental conditions is presented in Table 1. The overall Chi-square test result ($\chi^{2}=45.571$, p<0.001) indicates a significant effect of the experimental conditions on SA states, demonstrating that the experimental design successfully induces varying levels of SA.

Post hoc analysis revealed that the comparison between the LL and HH conditions showed the most significant difference ($\chi^{2}=44.41$, p<0.001), indicating that the HH condition was the most effective in inducing a low SA state. Additionally, both the LL vs. LH and LL vs. HL comparisons showed significant differences ($\chi^{2}=11.47$, p=0.00071; $\chi^{2}=11.17$, p=0.00083, respectively), suggesting that inducing either fatigue or stress alone also effectively reduces SA, though to a lesser degree than inducing both simultaneously. Furthermore, while no significant difference was observed between the LH and HL conditions ($\chi^{2}=0.00$, p=1.00), the comparisons of LH vs. HH ($\chi^{2}=10.60$, p=0.00113) and HL vs. HH ($\chi^{2}=11.12$, p=0.00086) confirmed that inducing both fatigue and stress simultaneously was significantly more effective than inducing either factor alone.

### 4.4. Impact of SA Variations on Typical Multimodal Physiological Features

The following analyses of multimodal physiological features reflect differences between high and low SA levels across all experimental conditions (Condition 1–Condition 4). These conditions were specifically designed to induce SA variations by manipulating levels of fatigue and stress, thus enabling a robust comparison of physiological differences associated with SA changes. The physiological feature variations reported in this section are therefore associated with SA level differences rather than being directly influenced by any single experimental condition.

#### 4.4.1. Eye Movement Patterns

**Figure 6:**
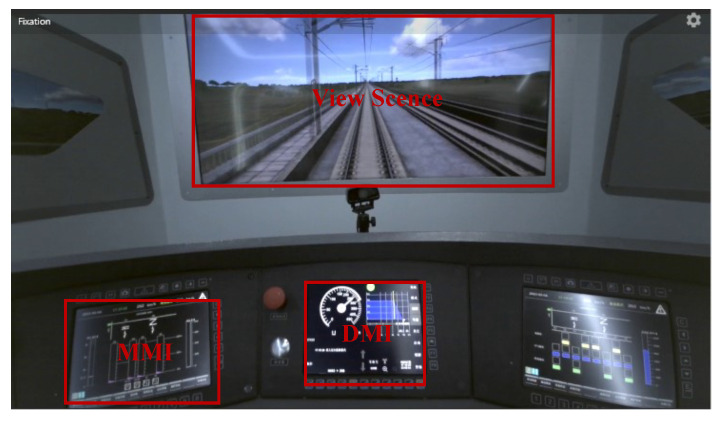
AOIs.

To analyze SA-related safety risks, we identified three Areas of Interest (AOIs): View Scene, MMI, and DMI (see Figure 6).

**Figure 7:**
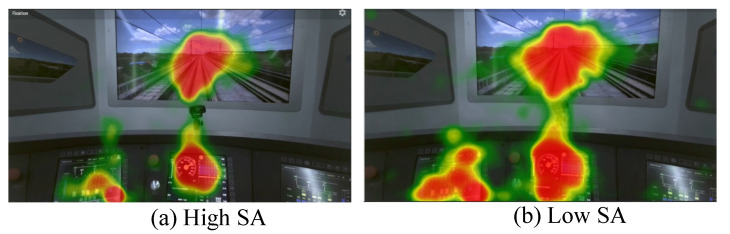
ET heatmaps at high and low SA levels.

The analysis of eye movement heatmaps (Figure 7) illustrates that variations in SA significantly influence attention distribution patterns. Under high SA level, participants primarily focus on the external environment (i.e., the visual field) while selectively attending to dashboard information. This selective attention enables participants to monitor critical external information efficiently, while only glancing at dashboard instruments when necessary, indicating high task efficiency and cognitive processing capacity. In contrast, under low SA level, participants’ attention shifted away from the external environment and became more reliant on the dashboard displays. This shift suggests that participants may experience higher cognitive load or fatigue, which in turn reduces their awareness of external environmental cues, leading to a decline in SA. The increased focus on dashboard information under low SA may reflect an attempt to compensate for the reduced external awareness, yet it ultimately hampers overall situational awareness.

#### 4.4.2. ECG Features

**Figure 8:**
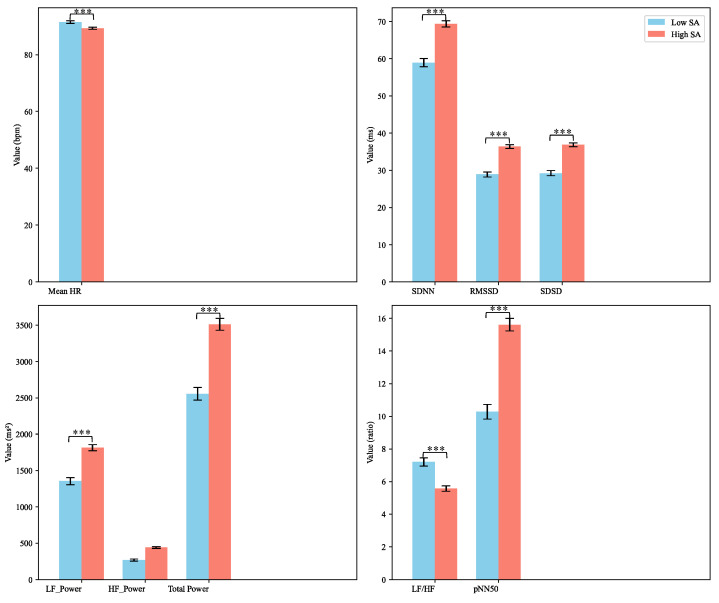
Effect of SA Variations on ECG Features. *** indicates *p* < 0.001.

The influence of SA variations on ECG signals was investigated using the Mann-Whitney U test, and the results are visualized in Figure 8. The analysis reveals that changes in SA levels significantly affect ECG signal characteristics. Under high SA conditions, participants exhibited a lower HR and increased HRV metrics, such as SDNN, RMSSD, SDSD, and pNN50. These findings suggest that the autonomic nervous system operates more efficiently, with enhanced parasympathetic nervous activity, indicating that participants are in a more relaxed and focused state. Conversely, under low SA conditions, heart rate was significantly higher, and HRV metrics showed a marked decrease. This reduction in HRV reflects a diminished capacity of the autonomic nervous system to regulate itself, suggesting that participants may be under higher levels of stress or cognitive load.

#### 4.4.3. EEG Features

**Figure 9:**
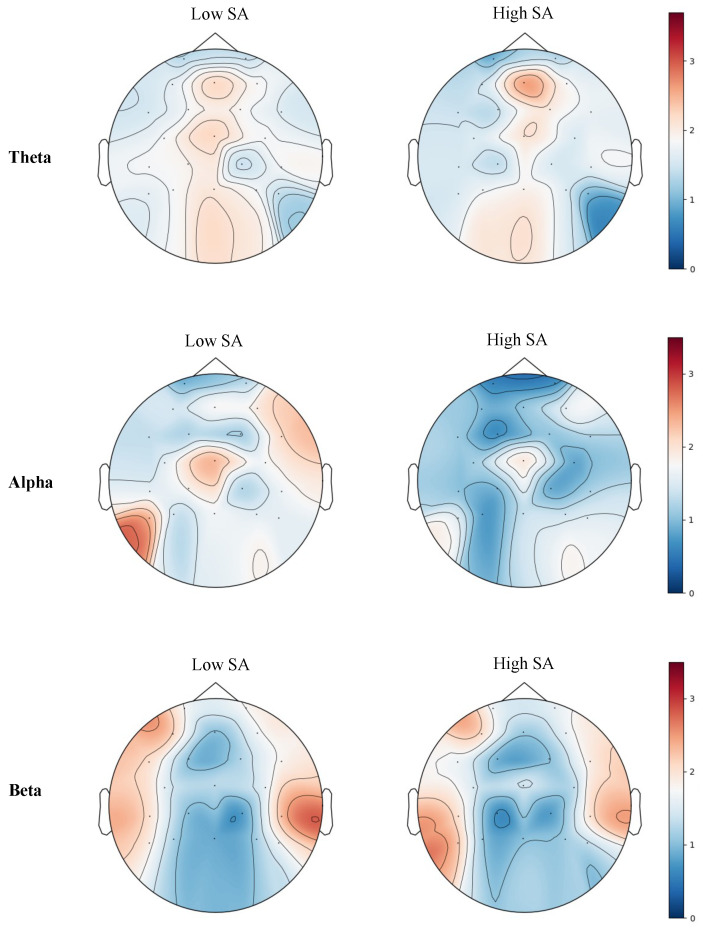
Effect of SA Variations on EEG Features.

The topographic maps of EEG PSD for three frequency bands (theta, alpha, and beta) under high and low SA levels are presented in Figure 9. Differences in PSD between high and low SA conditions were examined using the Wilcoxon signed-rank test. The results indicate that variations in SA levels significantly affect the electrical activity of different brain regions.

Under low SA level, a significant reduction in theta activity was observed in the frontal region (Wilcoxon signed-rank test statistic = 47.0, p=0.016), potentially reflecting heightened cognitive load and mental strain. Additionally, alpha activity in the frontal and parietal regions was more pronounced under low SA (Wilcoxon signed-rank test statistic = 48.0, p=0.020), suggesting that individuals may require more cognitive resources to cope with complex situations. In contrast, under high SA level, increased beta activity in the frontal and parietal regions (Wilcoxon signed-rank test statistic = 58.0, p=0.046) was noted, indicating greater task engagement and focus.

## 5. Discussion

In the context of high-speed train driving, this study developed an SA variation induction method tailored to this background and a more accurate SA labeling method. Based on these, the study explored how changes in SA in high-speed train driving affect multimodal physiological signals, including ET, ECG, and EEG. The findings demonstrate that variations in SA significantly impact these physiological markers, highlighting their potential for real-time SA monitoring in safety-critical environments.

The SA labeling method in this study was based on a combination of performance and subjective SA ratings, effectively distinguishing high and low SA levels. This approach combines the objectivity of performance with subjective SA assessments to establish the ground truth for labeling physiological data. The focus of future research should be on improving the accuracy of SA labeling for physiological signals, for example, by incorporating more objective measurement methods like SAGAT, to further enhance the accuracy of exploring the relationship between SA and physiological signal features.

In SA-related research, finding suitable SA measurement methods for the application context is crucial. This study emphasizes the importance of designing effective SA measurement methods tailored for high-speed train driving. Through statistical analysis, the experimental design in this study was validated, demonstrating that manipulating fatigue and stress successfully induced variations in SA. These results confirmed the effective induction of both mental states and SA state changes, providing a reliable data foundation for studying the relationship between SA and physiological signals in this context. Although low SA states can occur even under low-fatigue and low-stress conditions due to factors such as mind wandering, the SA measurement paradigm developed in this study offers a more reliable means of inducing SA variations. Mind wandering, a phenomenon where attention drifts away from the primary task, can lead to disengagement and reduced SA, as drivers may fail to process relevant external cues. Studies have shown that mind wandering impairs SA by diminishing the ability to respond to critical events promptly, especially in low-demand situations where perceptual load is minimal [57,58]. By incorporating controlled manipulations of fatigue and stress, the developed paradigm induces SA changes more consistently than would naturally occur, thereby reducing variability introduced by spontaneous internal cognitive shifts. This approach enhances data reliability, supporting more accurate SA assessments based on physiological features and offering a robust foundation for future research on multimodal physiological signals.

Eye movement analysis revealed that SA variations significantly influence attention distribution patterns. Under high SA level, participants focused more on the external environment and showed selective attention to dashboard information, demonstrating effective situational awareness and task efficiency. In contrast, low SA levels were associated with reduced attention to external cues and increased dependence on dashboard displays, suggesting higher cognitive load or fatigue. These findings align with previous research that links higher SA to better allocation of attention and more efficient information processing [22,59]. These results align with previous research linking eye movement patterns to SA, emphasizing the potential of ET data as a non-invasive, real-time indicator of SA level.

The ECG results show that SA variations impact HR and HRV metrics. Under high SA level, participants exhibited lower HR and increased HRV, indicating enhanced autonomic regulation [60] and a more relaxed state [61]. In low SA level, higher HR and reduced HRV reflect greater stress [62] and cognitive load [63]. These findings are consistent with the literature suggesting that physiological markers, such as HR and HRV, can be indicators of SA [64].

EEG analysis provided further insights into the neural correlates of SA. In low SA levels, a decrease in frontal theta activity was observed. Increased theta activity in high SA likely indicates enhanced memory and voluntary attention processes, as theta oscillations in the frontal cortex are associated with attention and working memory [51,52]. For alpha activity, a clear decrease was observed in high SA levels in the parietal and occipital regions, suggesting alpha desynchronization. Alpha desynchronization has been linked to increased activating influences from the reticular formation on the neocortex, indicating enhanced cortical processing and readiness [53,54]. In contrast, beta activity showed an increase in high SA levels in the parietal and occipital regions, reflecting greater task engagement and more efficient cognitive processing. Beta oscillations are associated with maintaining cognitive stability and task focus, which aligns with heightened SA [55,56]. These findings further validate the use of EEG signals to monitor SA states and provide insights into the neural mechanisms underlying SA.

Overall, our study demonstrates that variations in SA are reflected in distinct changes across multiple physiological modalities, including ET, ECG, and EEG. The integration of these signals can offer a comprehensive approach to real-time SA monitoring, with practical implications for enhancing safety and performance in high-speed train driving and other safety-critical domains.

### Limitations and Future Research Directions

This study has several limitations that should be noted, along with directions for future research to address these issues. First, the sample consisted of only 19 male train driver trainees, which may restrict the generalizability of the findings. This choice was due to the practical context in China, where nearly all high-speed train drivers are male, making male participants representative of the population. However, future studies should aim to include a more diverse sample in terms of experience and demographics to validate the results across different populations.

Second, while we utilized subjective stress ratings to confirm the effectiveness of stress induction, the lack of objective physiological measures for stress validation may limit the reliability of these findings. Subjective measures, though widely used, may not fully capture the physiological components of stress. Future studies could incorporate objective physiological stress indicators, such as HRV frequency-domain features or electrodermal activity, to provide a more comprehensive validation of stress induction. This addition would strengthen the robustness of the results by verifying the effectiveness of stress induction through both subjective and physiological metrics.

Additionally, this study focused on exploring the relationship between SA and a set of typical multimodal physiological features. While these features offer valuable insights, they may not capture the full complexity of SA states. For instance, the EEG analysis was restricted to three primary frequency bands (theta, alpha, and beta), chosen for their established associations with cognitive functions relevant to SA. However, this selection may limit the ability to capture other neural dynamics that could also play a role in SA variations. Future research could expand this scope by incorporating a broader range of physiological features, additional EEG frequency bands, or adopting a more comprehensive spectral analysis approach. Such enhancements would support a more complete understanding of SA dynamics and increase the applicability of these methods to other safety-critical domains beyond high-speed train driving.

To enhance the generalizability of these SA measurement methods, future studies should examine their effectiveness in real-world high-speed train driving environments and other safety-critical contexts. Expanding the range of physiological features beyond the typical metrics studied here could yield a more comprehensive understanding of SA dynamics. This may involve exploring additional physiological signals or employing advanced signal processing techniques to capture nuanced changes in SA states. By broadening the physiological indicators and refining real-time applications, researchers can improve the robustness and applicability of SA monitoring across diverse operational contexts.

Finally, real-time SA monitoring presents a promising direction for improving safety in high-stakes domains by allowing for proactive interventions based on operators’ real-time cognitive states. Although this study primarily focused on offline data analysis, the development of real-time applications would require addressing key challenges, such as computational efficiency, signal processing speed, and the robustness of SA assessments under varied operational conditions. Future research should explore advancements in wearable sensors, improved machine learning algorithms, and efficient signal processing techniques to support the feasibility of real-time SA monitoring. By incorporating robust machine learning methods, such as deep neural networks, researchers can potentially integrate multimodal physiological signals more effectively, thus enhancing real-time SA assessment. Additionally, improved algorithms for artifact removal and data preprocessing will be essential to ensure accuracy in real-time environments.

## Figures and Tables

**Table 1:** Distribution of SA samples under each experimental condition.

Experimental Condition	Low SA Samples	High SA Samples	Total Samples
LL	102	310	412
LH	151	271	422
HL	153	277	430
HH	207	234	441

## Data Availability

Restrictions apply to the availability of these data. Data were obtained from train driver trainees of Zhengzhou Railway Vocational and Technical College and are available from Wenli Dong with the permission of Zhengzhou Railway Vocational and Technical College.

## Appendix A. Adapted Mission Awareness Scale (MARS)

**Instructions:**

Please answer the following questions about the task you have just completed. Please select the option that best matches your experience and tick it.

**Questions:**

1. Please rate your ability to identify critical information (e.g., speed, fault events, traveling traffic, etc.) in this drive.
  □ very easy-able to identify all cues
  □ fairly easy-could identify most cues
  □ somewhat difficult-many cues hard to identify
  □ very difficult-had substantial problems identifying most cues
2. How well did you understand what was going on during the drive?
  □ very well-fully understood the situation as it unfolded
  □ fairly well-understood most aspects of the situation
  □ somewhat poorly-had difficulty understanding much of the situation
  □ very poorly-the situation did not make sense to me
3. How well could you predict what was about to occur next in the drive (e.g., speed changes, fault/driving events, etc.)?
  □ very well-could predict with accuracy what was about to occur
  □ fairly well-could make accurate predictions most of the time
  □ somewhat poorly-misunderstood the situation much of the time
  □ very poorly-unable to predict what was about to occur
4. How aware were you of how to best achieve your goals (follow speed tracking requirements; timely and correct handling of fault/driving events) during this drive?
  □ very aware-knew how to achieve goals at all times
  □ fairly aware-knew most of the time how to achieve mission goals
  □ somewhat unaware-was not aware of how to achieve some goals
  □ very unaware-generally unaware of how to achieve goals
5. How difficult-in terms of mental effort required-was it for you to identify critical information (e.g., speed, fault/driving events etc.) in this drive?
  □ very easy-could identify relevant cues with little effort
  □ fairly easy-could identify relevant cues, but some effort required
  □ somewhat difficult-some effort was required to identify most cues
  □ very difficult-substantial effort was required to identify relevant cues
6. How difficult-in terms of mental effort-was it to understand what was going on during this drive?
  □ very easy-understood what was going on with little effort
  □ fairly easy-understood events with only moderate effort
  □ somewhat difficult-hard to comprehend some aspects of situation
  □ very difficult-hard to understand most or all aspects of situation
7. How difficult-in terms of mental effort-was it to predict what was about to happen during this drive?
  □ very easy-little or no effort needed
  □ fairly easy-moderate effort required
  □ somewhat difficult-many projections required substantial effort
  □ very difficult-substantial effort required on most or all projections
8. How difficult-in terms of mental effort-was it to decide on how to best achieve your goals (follow speed tracking requirements; timely and correct handling of fault/driving events) during this exercise?
  □ very easy-little or no effort needed
  □ fairly easy-moderate effort required
  □ somewhat difficult-substantial effort needed on some decisions
  □ very difficult-most or all decisions required substantial effort

## References

[1] Strauch B. (2017). Investigating Human Error: Incidents, Accidents, and Complex Systems.

[2] Shappell S.A., Wiegmann D.A. (1996). US naval aviation mishaps, 1977–1992: Differences between single-and dual-piloted aircraft. Aviat. Space Environ. Med..

[3] Shappell S., Detwiler C., Holcomb K., Hackworth C., Boquet A., Wiegmann D.A. (2017). Human error and commercial aviation accidents: An analysis using the human factors analysis and classification system. Human Error in Aviation.

[4] Mathavara K., Ramachandran G. (2022). Role of human factors in preventing aviation accidents: An insight. Aeronautics-New Advances.

[5] Hamer R., Waterson P., Jun G.T. (2021). Human factors and nuclear safety since 1970–A critical review of the past, present and future. Saf. Sci..

[6] Meshkati N. (1991). Human factors in large-scale technological systems’ accidents: Three Mile Island, Bhopal, Chernobyl. Ind. Crisis Q..

[7] Ciani L., Guidi G., Patrizi G. (2022). Human reliability in railway engineering: Literature review and bibliometric analysis of the last two decades. Saf. Sci..

[8] Naderpour M., Nazir S., Lu J. (2015). The role of situation awareness in accidents of large-scale technological systems. Process Saf. Environ. Prot..

[9] Endsley M.R. (1995). Toward a theory of situation awareness in dynamic systems. Hum. Factors.

[10] Endsley M.R. (1995). A taxonomy of situation awareness errors. Hum. Factors Aviat. Oper..

[11] Naweed A. (2013). Psychological factors for driver distraction and inattention in the Australian and New Zealand rail industry. Accid. Anal. Prev..

[12] McLeod R.W., Walker G.H., Moray N. (2005). Analysing and modelling train driver performance. Appl. Ergon..

[13] Roth E., Multer J. (2009). Technology Implications of a Cognitive Task Analysis for Locomotive Engineers.

[14] Naweed A. (2014). Investigations into the skills of modern and traditional train driving. Appl. Ergon..

[15] Gugerty L. (2011). Situation awareness in driving. Handbook for Driving Simulation in Engineering, Medicine and Psychology.

[16] Salmon P.M., Stanton N.A., Young K.L. (2012). Situation awareness on the road: Review, theoretical and methodological issues, and future directions. Theor. Issues Ergon. Sci..

[17] Baysari M.T., Caponecchia C., McIntosh A.S., Wilson J.R. (2009). Classification of errors contributing to rail incidents and accidents: A comparison of two human error identification techniques. Saf. Sci..

[18] Zhang T., Yang J., Liang N., Pitts B.J., Prakah-Asante K., Curry R., Duerstock B., Wachs J.P., Yu D. (2023). Physiological measurements of situation awareness: A systematic review. Hum. Factors.

[19] Munir A., Aved A., Blasch E. (2022). Situational awareness: Techniques, challenges, and prospects. AI.

[20] Nguyen T., Lim C.P., Nguyen N.D., Gordon-Brown L., Nahavandi S. (2019). A review of situation awareness assessment approaches in aviation environments. IEEE Syst. J..

[21] Hasanzadeh S., Esmaeili B., Dodd M.D. (2018). Examining the relationship between construction workers’ visual attention and situation awareness under fall and tripping hazard conditions: Using mobile eye tracking. J. Constr. Eng. Manag..

[22] Moore K., Gugerty L. (2010). Development of a novel measure of situation awareness: The case for eye movement analysis. Proceedings of the Human Factors and Ergonomics Society Annual Meeting.

[23] Paletta L., Dini A., Murko C., Yahyanejad S., Schwarz M., Lodron G., Ladstätter S., Paar G., Velik R. Towards real-time probabilistic evaluation of situation awareness from human gaze in human-robot interaction. Proceedings of the Companion of the 2017 ACM/IEEE International Conference on Human-Robot Interaction.

[24] Van De Merwe K., Van Dijk H., Zon R. (2012). Eye movements as an indicator of situation awareness in a flight simulator experiment. Int. J. Aviat. Psychol..

[25] Liu W., Xue W. An analysis of situation awareness for the car cab display interface assessment based on driving simulation. Proceedings of the Electronics, Communications and Networks IV-Proceedings of the 4th International Conference on Electronics, Communications and Networks, CECNet2014.

[26] Wei H., Zhuang D., Wanyan X., Wang Q. (2013). An experimental analysis of situation awareness for cockpit display interface evaluation based on flight simulation. Chin. J. Aeronaut..

[27] Mehta R.K., Peres S.C., Shortz A.E., Hoyle W., Lee M., Saini G., Chan H.C., Pryor M.W. (2018). Operator situation awareness and physiological states during offshore well control scenarios. J. Loss Prev. Process Ind..

[28] Saus E.R., Johnsen B.H., Eid J., Riisem P.K., Andersen R., Thayer J.F. (2006). The effect of brief situational awareness training in a police shooting simulator: An experimental study. Mil. Psychol..

[29] Sun G., Wanyan X., Wu X., Zhuang D. The Influence of HUD Information Visual Coding on pilot’s Situational Awareness. Proceedings of the 2017 9th International Conference on Intelligent Human-Machine Systems and Cybernetics (IHMSC).

[30] Feng C., Liu S., Wanyan X., Chen H., Min Y., Ma Y. (2022). EEG feature analysis related to situation awareness assessment and discrimination. Aerospace.

[31] Kang Y., Liu F., Chen W., Li X., Tao Y., Huang W. (2024). Recognizing situation awareness of forklift operators based on eye-movement & EEG features. Int. J. Ind. Ergon..

[32] Yeo L.G., Sun H., Liu Y., Trapsilawati F., Sourina O., Chen C.H., Mueller-Wittig W., Ang W.T. Mobile EEG-based situation awareness recognition for air traffic controllers. Proceedings of the 2017 IEEE International Conference on Systems, Man, and Cybernetics (SMC).

[33] Crothers N., Sinha Y., Larson E.C., Scielzo S. (2022). Real-Time Situation Awareness Assessment for Pilots via Machine Learning: Constructing an Automated Classification System. MODSIM World.

[34] Fernandez Rojas R., Debie E., Fidock J., Barlow M., Kasmarik K., Anavatti S., Garratt M., Abbass H. (2019). Encephalographic assessment of situation awareness in teleoperation of human-swarm teaming. Proceedings of the Neural Information Processing: 26th International Conference, ICONIP 2019.

[35] Zhou F., Yang X.J., De Winter J.C. (2021). Using eye-tracking data to predict situation awareness in real time during takeover transitions in conditionally automated driving. IEEE Trans. Intell. Transp. Syst..

[36] Li R., Lan Z., Cui J., Sourina O., Wang L. EEG-based recognition of driver state related to situation awareness using graph convolutional networks. Proceedings of the 2020 International Conference on Cyberworlds (CW).

[37] Li Q., Ng K.K., Simon C., Yiu C.Y., Lyu M. (2023). Recognising situation awareness associated with different workloads using EEG and eye-tracking features in air traffic control tasks. Knowl. Based Syst..

[38] Endsley M.R. (2020). The divergence of objective and subjective situation awareness: A meta-analysis. J. Cogn. Eng. Decis. Mak..

[39] Foody G.M. (2024). Ground Truth in Classification Accuracy Assessment: Myth and Reality. Geomatics.

[40] De Winter J.C., Eisma Y.B., Cabrall C.D., Hancock P.A., Stanton N.A. (2019). Situation awareness based on eye movements in relation to the task environment. Cogn. Technol. Work..

[41] Smith M.R., Chai R., Nguyen H.T., Marcora S.M., Coutts A.J. (2019). Comparing the effects of three cognitive tasks on indicators of mental fatigue. J. Psychol..

[42] Hachard B., Noé F., Ceyte H., Trajin B., Paillard T. (2020). Balance control is impaired by mental fatigue due to the fulfilment of a continuous cognitive task or by the watching of a documentary. Exp. Brain Res..

[43] Sikander G., Anwar S. (2018). Driver fatigue detection systems: A review. IEEE Trans. Intell. Transp. Syst..

[44] Bobade P., Vani M. Stress detection with machine learning and deep learning using multimodal physiological data. Proceedings of the 2020 Second International Conference on Inventive Research in Computing Applications (ICIRCA).

[45] Matthews M.D. (2002). Situation Awareness in a Virtual Environment: Description of a Subjective Assessment Scale.

[46] Lo J.C., Sehic E., Brookhuis K.A., Meijer S.A. (2016). Explicit or implicit situation awareness? Measuring the situation awareness of train traffic controllers. Transp. Res. Part F.

[47] Baek H.J., Cho C.H., Cho J., Woo J.M. (2015). Reliability of ultra-short-term analysis as a surrogate of standard 5-min analysis of heart rate variability. Telemed. e-Health.

[48] Laborde S., Mosley E., Thayer J.F. (2017). Heart rate variability and cardiac vagal tone in psychophysiological research–recommendations for experiment planning, data analysis, and data reporting. Front. Psychol..

[49] Shaffer F., Ginsberg J.P. (2017). An overview of heart rate variability metrics and norms. Front. Public Health.

[50] Kästle J.L., Anvari B., Krol J., Wurdemann H.A. (2021). Correlation between Situational Awareness and EEG signals. Neurocomputing.

[51] Sauseng P., Klimesch W. (2008). What does phase information of oscillatory brain activity tell us about cognitive processes?. Neurosci. Biobehav. Rev..

[52] Kahana M.J., Seelig D., Madsen J.R. (2001). Theta returns. Curr. Opin. Neurobiol..

[53] Klimesch W. (2012). Alpha-band oscillations, attention, and controlled access to stored information. Trends Cogn. Sci..

[54] Pfurtscheller G., Da Silva F.L. (1999). Event-related EEG/MEG synchronization and desynchronization: Basic principles. Clin. Neurophysiol..

[55] Engel A.K., Fries P. (2010). Beta-band oscillations—Signalling the status quo?. Curr. Opin. Neurobiol..

[56] Hanslmayr S., Staudigl T., Fellner M.C. (2012). Oscillatory power decreases and long-term memory: The information via desynchronization hypothesis. Front. Hum. Neurosci..

[57] Tinella L., Koppel S., Lopez A., Caffò A.O., Bosco A. (2022). Associations between personality and driving behavior are mediated by mind-wandering tendency: A cross-national comparison of Australian and Italian drivers. Transp. Res. Part F.

[58] Lopez A., Caffò A.O., Tinella L., Bosco A. (2023). The four factors of mind wandering questionnaire: Content, construct, and clinical validity. Assessment.

[59] Cak S., Say B., Misirlisoy M. (2020). Effects of working memory, attention, and expertise on pilots’ situation awareness. Cogn. Technol. Work..

[60] Von Borell E., Langbein J., Després G., Hansen S., Leterrier C., Marchant J., Marchant-Forde R., Minero M., Mohr E., Prunier A. (2007). Heart rate variability as a measure of autonomic regulation of cardiac activity for assessing stress and welfare in farm animals—A review. Physiol. Behav..

[61] Hernando D., Roca S., Sancho J., Alesanco Á., Bailón R. (2018). Validation of the apple watch for heart rate variability measurements during relax and mental stress in healthy subjects. Sensors.

[62] Kim H.G., Cheon E.J., Bai D.S., Lee Y.H., Koo B.H. (2018). Stress and heart rate variability: A meta-analysis and review of the literature. Psychiatry Investig..

[63] Solhjoo S., Haigney M.C., McBee E., van Merrienboer J.J., Schuwirth L., Artino A.R., Battista A., Ratcliffe T.A., Lee H.D., Durning S.J. (2019). Heart rate and heart rate variability correlate with clinical reasoning performance and self-reported measures of cognitive load. Sci. Rep..

[64] Rajendran A., Kebria P.M., Mohajer N., Khosravi A., Nahavandi S. Machine learning based prediction of situational awareness in pilots using ecg signals. Proceedings of the 2021 IEEE Symposium Series on Computational Intelligence (SSCI).

